# Venous thrombectomy using the InThrill Thrombectomy System: preliminary experiences

**DOI:** 10.1186/s42155-024-00498-8

**Published:** 2024-11-23

**Authors:** Kevin M. McElroy, David S. Shin, Matthew Abad-Santos, Eric J. Monroe, Jeffrey Forris Beecham Chick

**Affiliations:** 1 Section of Interventional Radiology, Department of Radiology, University of Washington, 1959 Northeast Pacific Street, Seattle, WA 98195 USA; 2 Division of Vascular and Interventional Radiology, Department of Radiology, University of Southern California, 1500 San Pablo Street, Los Angeles, CA 90033 USA; 3 Section of Vascular and Interventional Radiology, Department of Radiology, University of Wisconsin, 600 Highland Avenue, Madison, WI 53792 USA; 4 The Deep Vein Institute, University of Washington, 1959 Northeast Pacific Street, Seattle, WA 98195 USA

Mechanical thrombectomy provides endovascular removal of venous thrombus [1–3]. Use of large-bore thrombectomy devices (13-French) has been previously described [2, 3]. The small-bore 8-French InThrill Thrombectomy System (Inari Medical; Irvine, CA) is indicated for “*small venous thrombectomy in 4-10-mm vessels*” according to the manufacturer. This report describes initial use of the InThrill Thrombectomy System for the treatment of symptomatic deep vein thrombosis in various locations. *Institutional review board approval was obtained for preparation of this report*.

Eleven patients (six males and five females; mean age 49.6 ± 16.1 years [range: 39–72 years]) presented with symptomatic venous occlusive disease and underwent InThrill-mediated mechanical thrombectomy. Mean duration from imaging-detected thrombosis to thrombectomy was 19.1 ± 27.7 days (range: 3–77 days), with 9/12 (75%) patients presenting with acute venous thrombosis (< 14 days) and 3/12 (25%) presenting with chronic venous thrombosis (≥ 14 days). Thrombosis locations included: portomesenteric venous system (*n* = 3), brachiocephalocaval veins (*n* = 2), iliocaval stent constructs (*n* = 2), internal jugular vein (*n* = 1), arteriovenous graft (*n* = 1), renal vein (*n* = 1), and iliofemoral vein (*n* = 1). The dedicated InThrill Sheath was used in 7/11 (63.6%) patients. Given the length constraint, the InThrill Sheath was not used in portomesenteric venous interventions (*n* = 3) and in a procedure where the 20-French Protrieve Sheath (Inari Medical) was used (*n* = 1) [4]. Mean vessel diameter of the thrombosed vein was 11.7 *±* 2.7-mm (range: 6-14-mm). Access sites, into which the InThrill Catheter was placed, included internal jugular vein (*n* = 5), brachial vein (*n* = 2), femoral vein (*n* = 2), common femoral vein (*n* = 1), and popliteal vein (*n* = 1). All portomesenteric interventions (*n* = 3) utilized a transjugular approach via left jugular vein access. One-to-three thrombectomy passes were performed, with < 25 mL blood loss, per patient. Technical success, described as improvement in in-line venous flow after InThrill-mediated thrombectomy, was achieved in 10/11 (90.9%) patients. One (9.1%) patient with septic thrombophlebitis of the internal jugular vein failed thrombectomy. Additional thrombectomy attempts were not made to limit potential septic thromboembolic adverse events. Additional supplementary thrombectomy devices were utilized in 4/11 (36.3%) patients including the AngioJet Peripheral Thrombectomy System (Boston Scientific; Marlborough, MA) (*n* = 3), ClotTriever Thrombectomy System (Inari Medical) (*n* = 1), T20 FlowTriever System (Inari Medical) (*n* = 1), T16 FlowTriever System (Inari Medical) (*n* = 1), and CAT12 Lightning Aspiration Catheter (Penumbra; Alameda, CA) (*n* = 1), based on operator preference. Venous stent reconstruction was performed in 6/11 (54.5%) patients. Clinical success, described as symptomatic improvement, was achieved in all patients where technical success was achieved. There were no immediate adverse events. Six (54.5%) patients were maintained on post-procedural anticoagulation and antiplatelet agents, four (36.4%) on anticoagulation alone, and one (9.1%) on neither anticoagulation nor antiplatelet agents. Two (18.1%) patients underwent additional future thrombectomy.

Patient 1 (iliocaval stent thrombectomy) (Fig. 1): A 53-year-old male, who underwent prior inferior vena cava filter removal with caval and bilateral iliocaval recanalization and stent reconstruction, presented with recurrent left lower extremity swelling. Left lower extremity venography demonstrated iliocaval and iliofemoral stent thrombosis (Fig. 1A). InThrill-mediated thrombectomy of the thrombosed stent construct was performed via left femoral vein access (Fig. 1B). The stent construct was then relined using two 14-mm x 100-mm, one 14-mm x 80-mm, and one 12-mm x 150-mm Abre Venous Stents (Medtronic; Minneapolis, MN). Completion venography demonstrated restored brisk in-line flow (Fig. 1C).

**Fig. 1:**
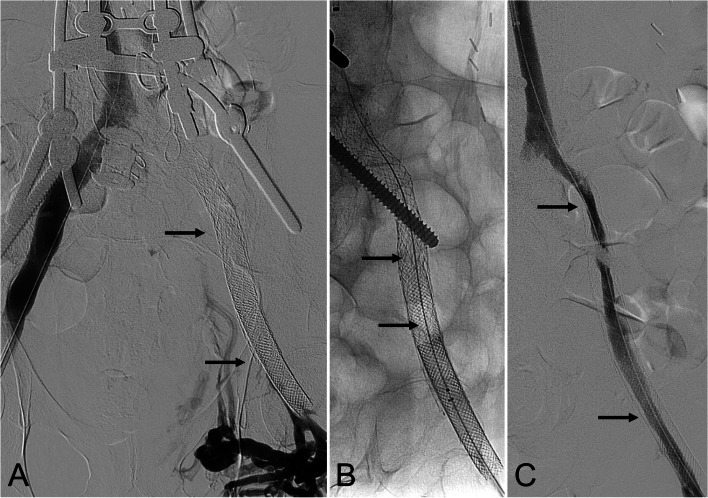
*53-year-old male with inferior vena cava filter-associated thrombosis status post filter removal with recanalization and stent reconstruction with recurrent left lower extremity swelling*. **A** Bilateral lower extremity ascending venography demonstrating thrombosis of the left iliofemoral and iliocaval stent construct (arrows). **B** Thrombectomy was performed using the InThrill Catheter (arrows). **C** After thrombectomy, there was restoration of in-line flow along the entire stent construct (arrows), which was then relined using two 14-mm x 100-mm, one 14-mm x 80-mm, and one 12-mm x 150-mm Abre stents (not shown)

Patient 2 (renal vein thrombectomy) (Fig. 2): A 57-year-old female with metastatic cervical carcinoma presented with right lower extremity deep vein thrombosis and suspected tumor thrombus in the left renal vein (Fig. 2A). Following right iliofemoral thrombectomy and stent reconstruction, left renal venography demonstrated renal vein thrombosis (Fig. 2B). The Protrieve Sheath was deployed into the suprarenal inferior vena cava via right internal jugular venous access [4]. InThrill-mediated thrombectomy of the left renal vein was then performed via left common femoral vein access (Fig. 2C). Completion venography demonstrated restored in-line flow (Fig. 2D). Pathologic analysis of removed thrombus demonstrated large areas of necrotic cells concerning for neoplasm, presumably from the cervical mass.

**Fig. 2:**
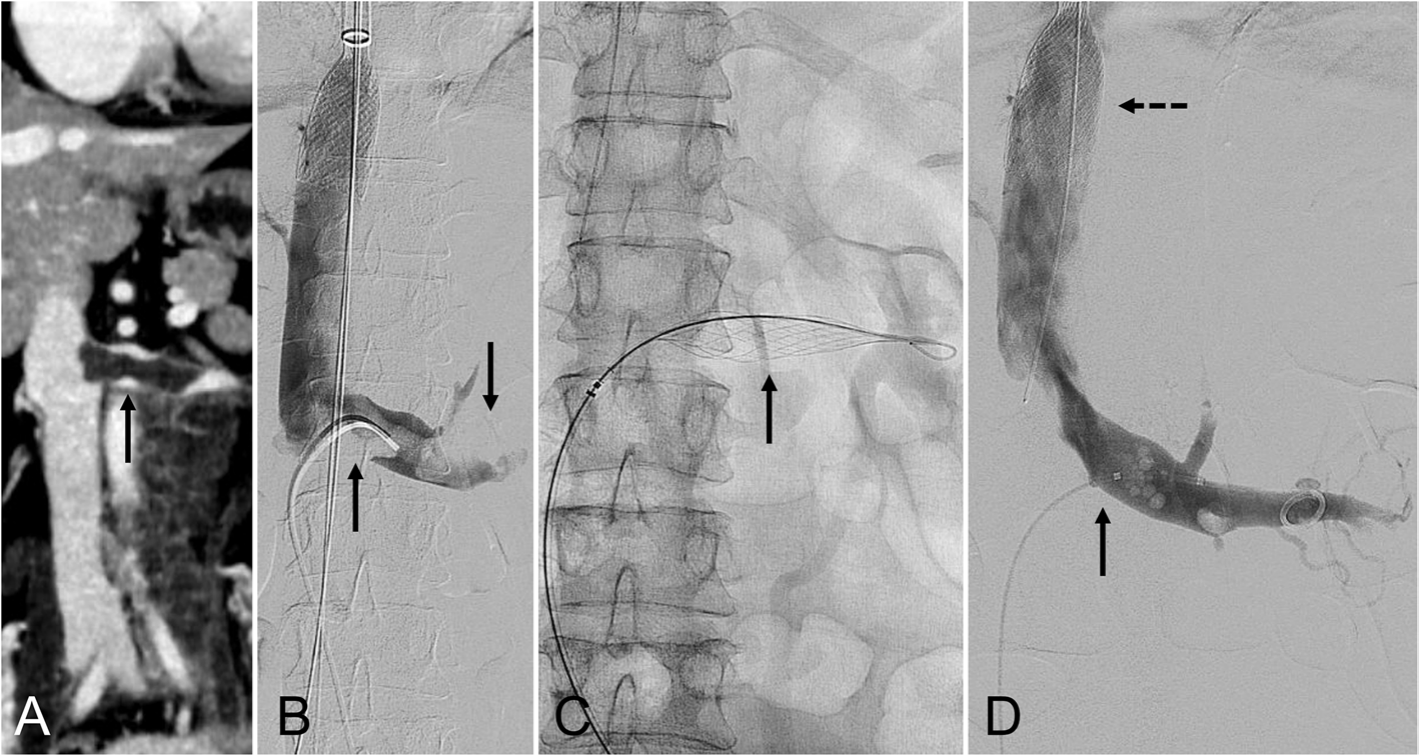
*57-year-old female with metastatic cervical carcinoma presented with right lower extremity deep vein thrombosis and suspected tumor thrombus in the left renal vein.* **A** Coronal computed tomographic venography of the abdomen and pelvis demonstrating left renal vein thrombosis (solid arrow). **B** Left renal venography confirmed left renal vein thrombosis (solid arrows). **C** Thrombectomy was performed using the InThrill Catheter (solid arrow). **D** After thrombectomy, there was brisk in-line flow along the left renal vein (solid arrow) with minimal filling defects. The ProTrieve Sheath (dashed arrow) was deployed during the thrombectomy to capture any embolic materials. Pathologic analysis of removed thrombi, from the ProTrieve Sheath, demonstrated large areas of necrotic and atypical cells concerning for neoplasm, presumably related to a cervical tumor (not shown)

Patient 3 (splenoportal thrombectomy) (Fig. 3): A 60-year-old non-cirrhotic female with leukocytoclastic vasculitis presented with abdominal pain and splenoportal thrombosis. Management with anticoagulation failed to resolve symptoms. Transjugular-approach portal venography demonstrated splenoportal thrombosis (Fig. 3A). Following a catheter-directed infusion of 3 mg of tissue plasminogen activator (Genentech; South San Francisco, CA), InThrill-mediated thrombectomy of the splenoportal venous segments was performed (Fig. 3B). A transjugular intrahepatic portosystemic shunt was created using a 10-mm x 8-cm (covered) x 2-cm (uncovered) Viatorr CX (W. L. Gore & Associates; Flagstaff, AZ) post-dilated to 8-mm. Given the extent of splenoportal thrombosis, additional thrombectomy was performed with the AngioJet Peripheral Thrombectomy System, T16 FlowTriever System, and the CAT12 Lightning Aspiration Catheter. Completion portal venography demonstrated restored in-line flow (Fig. 3C).

**Fig. 3:**
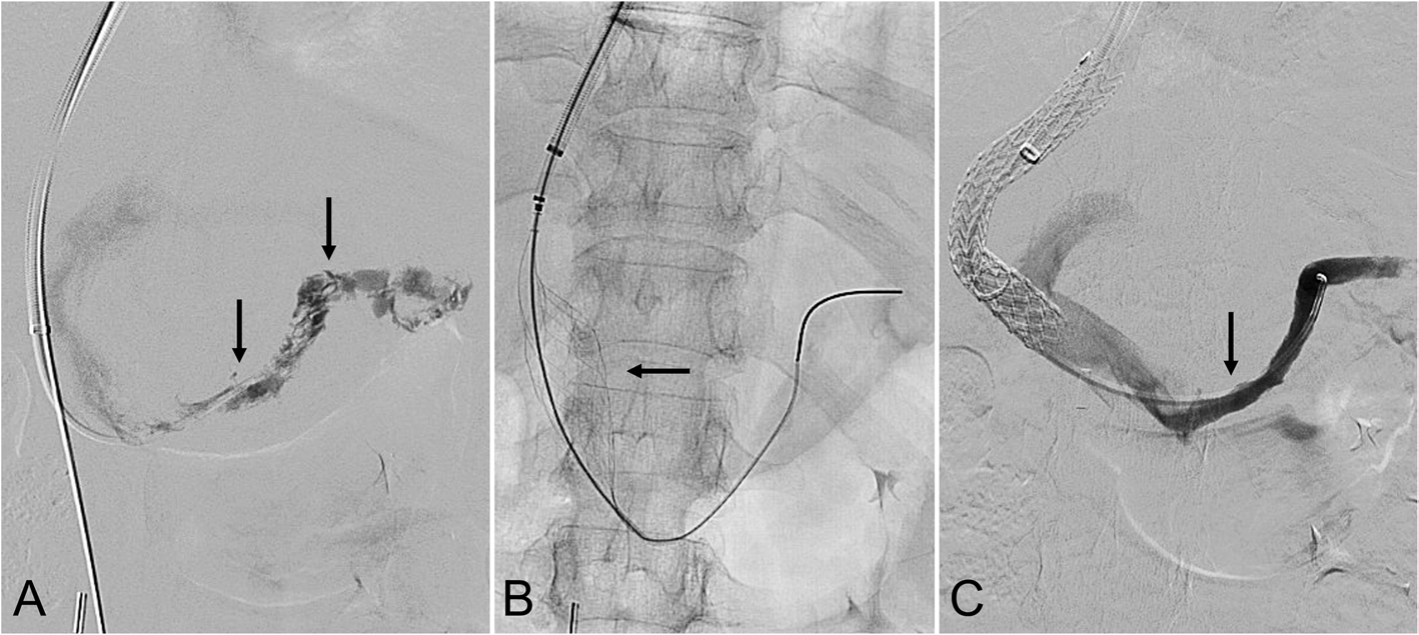
*60-year-old non-cirrhotic female presenting with leukocytoclastic vasculitis*,* abdominal pain*,* and splenoportal thrombosis*. **A** Splenoportal venography demonstrating thrombosis (arrows). **B** Thrombectomy was performed using the InThrill Catheter (arrow), which was pulled into the transjugular portal access sheath. **C** After thrombectomy and transjugular intrahepatic portosystemic shunt creation, there was restored in-line patency from the splenic vein (arrow) to the right atrium

This report describes preliminary experiences using the small-bore 8-French InThrill Thrombectomy System for the treatment of symptomatic deep vein thrombosis in various locations. The large-bore 13-French ClotTriever Thrombectomy System has been shown to be efficacious and safe for caval and lower extremity venous thrombectomy, and endovascular tissue sampling [2, 3]. However, use in small-diameter veins < 6 mm is not recommended. Conceptually similar to the ClotTriever Thrombectomy System, the InThrill Thrombectomy System features an over-the-wire coring element on a 65-cm catheter designed to engage, capture, and extract thrombi in smaller vessels. A short single-piece coring element/basket allows for use in venous segments where the longer ClotTriever coring element and capture basket length may be prohibitive. This series suggests that the InThrill Thrombectomy System may serve as a useful device for mechanical thrombectomy in smaller veins of various systems, including the splanchnic veins and thoracic and pelvic central veins. Technical limitations include the device length unable to reach thrombi remote from the vascular access site, the short length of the dedicated funnel sheath limiting its use to relatively superficial points of vascular access, and the comparatively low volume basket for thrombus capture. Further studies are warranted to establish the venous anatomy most amenable to InThrill-mediated thrombectomy.

## References

[1] Lijfering W, Rosendaal F, Cannegieter S. Risk factors for venous thrombosis – current understanding from an epidemiological point of view. Br J Haematol. 2010;149(6):824–33. 20456358 10.1111/j.1365-2141.2010.08206.x

[2] Benarroch-Gampel J, Pujari A, et al. Technical success and short-term outcomes after treatment of lower extremity deep vein thrombosis with the ClotTriever system: a preliminary experience. J Vasc Surg Venous Lymphat Disord. 2020;8(2):174–81. 31843476 10.1016/j.jvsv.2019.10.024

[3] Greenberg C, Shin D, et al. Endovascular tissue sampling using the ClotTriever Thrombectomy System: Histopathologic Analysis in 26 consecutive patients. Cardiovasc Intervent Radiol. 2022;45(6):898–901. 35364721 10.1007/s00270-022-03128-9 PMC8975446

[4] Greenberg C, Shin DS, Verst L, Monroe EJ, Bertino FJ, Abad-Santos M, Chick JFB. Protrieve Sheath embolic protection during venous thrombectomy: early experience in seventeen patients. CVIR Endovasc. 2024;7(1):74. 39382712 10.1186/s42155-024-00484-0 PMC11479621

